# P-404. A nationwide survey of Infection Prevention and Control and High-level Disinfection and Sterilization practices in the Dominican Republic

**DOI:** 10.1093/ofid/ofae631.605

**Published:** 2025-01-29

**Authors:** Elianet Castillo, Rita A Rojas-Fermin, Yeison Reyes, Claudia Blanco, Antonio M Villegas, Alfredo J Mena Lora

**Affiliations:** CEDIMAT/CEMDOE, Santo Domingo, Distrito Nacional, Dominican Republic; Hospital General de la Plaza de la Salud, Santo Domingo, Distrito Nacional, Dominican Republic; Hospital General de la Plaza de la Salud, Santo Domingo, Distrito Nacional, Dominican Republic; SDI, Santo Domingo, Distrito Nacional, Dominican Republic; CEDIMAT, Santo Domingo, Distrito Nacional, Dominican Republic; University of Illinois Chicago, Chicago, Illinois

## Abstract

**Background:**

Healthcare-Associated Infections (HAI) cause significantly morbidity and mortality worldwide. Infection Prevention and Control Programs (IPC) have proven to be effective in reducing HAIs. In low- and middle-income countries, 15% of patients in acute care hospitals acquire a hospital acquired infection (HAI) compared to 7% in high-income countries. Strong IPC can reduce HAIs and the spread of antimicrobial resistance (AMR). We seek to characterize IPC capabilities and practices in the Dominican Republic (DR).

**Figure 1:**
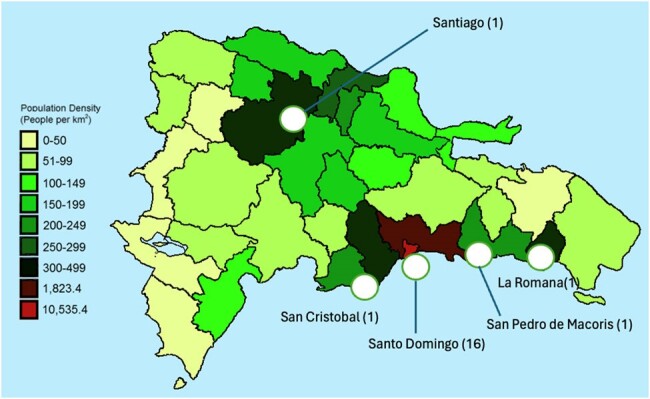
Population density of the Dominican Republic and geographic distrubution of hospitals surveyed

**Methods:**

We performed an anonymous survey of infectious diseases (ID) specialists in acute care hospitals in the DR. A survey based on the CDC Infection Control Assessment and Response (ICAR) tool for general infection and control was distributed via professional society listervs between March 4 and April 24, 2024. Data was tabulated and descriptive statistics performed.

**Figure:**
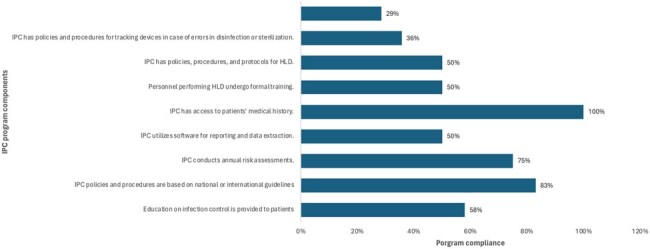

**Figure 2:** Components of Infection Prevention and Control programs in the Dominican Republic

**Results:**

A total of 20 hospitals responded to our survey, representing 16 facilities in Santo Domingo, and one in Santiago, San Cristobal, La Romana, and San Pedro de Macoris respectively (Figure 1). Of the 20 facilities, 60% (12) reported having an IPC Program, 55% (11) had a dedicated multidisciplinary IPC committee, and 90% had access to an IPC expert such as an ID physician (Figure 2). Education to patients was provided in 58% of hospitals, 83% set policies and procedures based on national or international guidelines, 75% conduct annual risk assessments, and 50% utilize software for data reporting and extraction. All IPC programs reported access to the medical record. Fourteen centers reported data on high-level disinfection and sterilization (HLD), of which 50% had formal training for staff, 50% reported having HLD specific policies, 35.7% track devices for errors, and 28.5% have a reporting process for device-related infections to public health authorities.

**Conclusion:**

Our survey provides an overview of the current landscape of IPC in the DR. While there has been significant progress in standardizing IPC practices, our findings indicate that nearly half of the surveyed hospitals do not have formal IPC programs. Notably, existing IPC programs often lack formal IPC committees, device tracking, data extraction, and infection reporting mechanisms. Addressing these gaps may help recude HAIs and curb AMR.

**Disclosures:**

**Rita A. Rojas-Fermin, MD,FIDSA**, Gilead: Advisor/Consultant|Pfizer: Advisor/Consultant|Pfizer: Honoraria

